# Research Progress in Enzyme Biofuel Cells Modified Using Nanomaterials and Their Implementation as Self-Powered Sensors

**DOI:** 10.3390/molecules29010257

**Published:** 2024-01-03

**Authors:** Lili Cao, Juan Chen, Jingyu Pang, Hongjie Qu, Jiaren Liu, Jinling Gao

**Affiliations:** College of Science, Heilongjiang Bayi Agricultural University, Daqing 163319, China; 15547002901@163.com (J.C.); jyp0963@163.com (J.P.); qhjsxm@163.com (H.Q.); liujiaren@byau.edu.cn (J.L.); gjlscl@sina.com (J.G.)

**Keywords:** enzyme biofuel cells (EBFCs), nanomaterials in EBFCs, chemical-to-electrical energy conversion, self-powered sensors, sustainable energy technology

## Abstract

Enzyme biofuel cells (EBFCs) can convert chemical or biochemical energy in fuel into electrical energy, and therefore have received widespread attention. EBFCs have advantages that traditional fuel cells cannot match, such as a wide range of fuel sources, environmental friendliness, and mild reaction conditions. At present, research on EBFCs mainly focuses on two aspects: one is the use of nanomaterials with excellent properties to construct high-performance EBFCs, and the other is self-powered sensors based on EBFCs. This article reviews the applied nanomaterials based on the working principle of EBFCs, analyzes the design ideas of self-powered sensors based on enzyme biofuel cells, and looks forward to their future research directions and application prospects. This article also points out the key properties of nanomaterials in EBFCs, such as electronic conductivity, biocompatibility, and catalytic activity. And the research on EBFCs is classified according to different research goals, such as improving battery efficiency, expanding the fuel range, and achieving self-powered sensors.

## 1. Introduction

With the development of self-powered sensor technology, nanomaterials have attracted widespread attention in the field of enzyme-based biofuel cells (EBFCs) [1,2]. The rapid advancement in this research domain strives to realize autonomous sensor systems, paving the way for innovative applications in wireless sensor networks, mobile devices, and wearable technologies. Enzyme-bioful-cell-based self-powered sensors not only demonstrate a high energy conversion efficiency but also offer distinctive benefits, courtesy of nanomaterials that are synthesized and incorporated at both the micro- and nanoscales [3,4].

Enzyme biofuel cells offer several key advantages: First and foremost, they are capable of efficiently harnessing renewable biological and abiotic components, such as sugars, alcohols, organic acids, hydrogen, and their mixtures, as fuel sources [5,6,7]. Second, enzymatic biofuel cells are fundamentally pollution-free. They operate effectively in water with a pH value close to 7 and under normal temperature and pressure conditions, and their components are biodegradable. Third, in principle, biological enzymes can be produced cost-effectively through genetic engineering and fermentation processes. Fourth, these biological enzymes exhibit high reaction selectivity and biocatalytic activity. Fifth, enzyme biofuel cells have good biocompatibility and can be implanted into the human body under certain conditions and provide power for other implanted electronic devices [8]. Considering these aforementioned advantages, along with the escalating demand for clean and sustainable energy sources and the swift advancement of implantable electronic devices in recent years, a substantial number of researchers have been motivated to engage in extensive studies on enzymatic biofuel cells. Consequently, enzymatic biofuel cells are introducing novel opportunities in the development of biosensors.

As a green and renewable energy conversion technology, enzymatic biofuel cells have become an important research direction in the field of self-powered sensors [9,10,11]. Its ability to utilize biocatalysts (enzymes) to catalyze fuel oxidation on the electrode surface enables it to exhibit excellent performance in environmentally friendly sensors. The introduction of nanomaterials provides finer control and enhanced performance for this technology [12,13].

This review is dedicated to methodically summarizing the current developments and applications of nanomaterials in the realm of self-powered sensors based on enzyme biofuel cells. Initially, it explores the fundamental principles of enzymatic biofuel cells, with a particular emphasis on their significance in energy conversion and sensor technology. The review then progresses to examine the crucial role of nanomaterials in the construction of enzymatic biofuel cells. This includes an in-depth analysis of how nanomaterials enhance enzyme activity, electron transfer efficiency, and biocompatibility. Moreover, the review provides a comprehensive discussion of the practical applications of nanomaterials in self-powered sensors, spanning diverse domains such as environmental monitoring, medical diagnostics, and wearable technologies. Compared with other works, this review mainly focuses on the design and application of different types of nanocatalytic systems in self-powered sensors. This article mainly introduces the working mechanism and performance evaluation methods of self-powered sensors and outlines the research progress and application prospects of self-powered sensors. This review synthesizes these aspects more fully and proposes some future challenges and opportunities.

By conducting a thorough analysis and compilation of the existing literature, this review aims to furnish researchers with an all-encompassing understanding of the most recent advancements and future trajectories in the use of nanomaterials for self-powered sensors based on enzyme biofuel cells. Progress in this area is poised to catalyze further innovations in self-powered sensor technology and is anticipated to unlock new horizons in the development of sustainable energy sources and autonomous sensing technologies.

## 2. Enzyme Biofuel Cells

In an effort to surmount the operational limitations of microbial fuel cells (MFCs), researchers have introduced enzymatic biofuel cells as an alternative class of biofuel cells, as illustrated in Table 1. Within these systems, biocatalysts, specifically oxidoreductases, are isolated and purified from living organisms. These biocatalysts are then brought into contact with the electrodes of the biofuel cell components to oxidize the target fuel, thereby facilitating energy generation. Enzymatic biofuel cells employ enzymes to catalyze the oxidation of fuel at the anode surface, resulting in a notably higher output power density compared to traditional MFCs. However, they are limited in usage conditions due to incomplete fuel oxidation and a short service life [14,15,16]. The catalytic activity of enzymes is a key factor, and enzymes are sensitive chemicals that require specific reaction conditions. In EBFC, enzymes oxidize the fuel and transfer electrons to the anode, where the biocatalyst participates in the reduction of the oxidant [17,18].

For most redox enzymes, direct electron transfer (DET) remains a challenge, especially between the redox center and the electrode [19]. In order to solve the problems of low power density and poor biocompatibility faced by EBFCs, researchers modified the electrodes with functionalized enzymes to improve performance [20,21]. This approach utilizes a coated composite biocatalytic layer that enables electrons to rapidly shuttle between the enzyme’s active site and the electrode surface, allowing for the development of a variety of enzyme biofuel cells with a high power density. Therefore, enzymatic biofuel cells have broad application prospects in wearable devices, implantable electronic devices, and other fields [22,23]. In addition, EBFCs have the advantages of mild operating conditions and a wide source of enzyme catalysts and are suitable for powering organs that can be implanted in the human body [24], so they have received increasing attention.

**Table 1:** Comparison of enzyme fuel cells and other fuel cells [25].

Fuel Cell Type	Fuel	Catalyst	Advantage	Disadvantage
Enzyme fuel cell	Organic matter, such as methanol, sugar, etc.	Enzyme	Green, safe, environmentally friendly, sustainable, high biocompatibility, suitable for wearable or implantable devices	Low energy density, low power density, poor stability, low voltage
Microbial fuel cell	Organic matter, such as wastewater, sludge, etc.	Microorganism	Can use waste to generate electricity, reduces pollution, and has low cost	Slow mass transfer rate, low electron transfer efficiency, low power density, and low voltage
Direct methanol fuel cell	Methanol	Precious metals such as platinum	Methanol is easy to store and has high energy density, making it suitable for use in portable equipment	Methanol permeates easily, reduces voltage, has high cost of precious metals, and causes environmental pollution
Hydrogen–oxygen fuel cell	Hydrogen	Precious metals such as platinum	No pollution, high energy conversion efficiency, suitable for use in cars or spacecrafts	Hydrogen storage is difficult, has poor safety, and the cost of precious metals is high

### 2.1. The Structure of Enzyme Biofuel Cells

A fuel cell is a device that is designed to convert chemical energy directly into electrical energy. Distinct from this, a biofuel cell is a specialized type of fuel cell that employs biological catalysts for its operation. In a conventional fuel cell, an oxidation reaction takes place at the anode, generating H^+^ ions (protons) and electrons. These electrons travel through an external circuit, providing electrical power, while the H^+^ ions migrate across a semipermeable membrane to the cathode. At the cathode, these H^+^ ions combine with oxygen, typically from the air, to form water, completing the chemical process [26,27].

**Figure 1:**
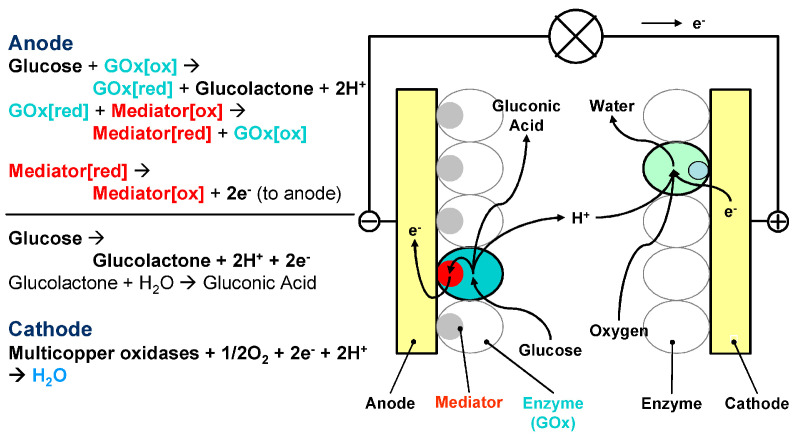
Schematic diagram of the working principle and structure of enzyme biofuel cells [31].

Biofuel cells work and are similar to traditional fuel cells, with two electrodes (anode and cathode), at least one of which contains a biocatalyst in the form of enzymes or microorganisms that use common organic compounds as fuel [28,29], as shown in Figure 1. The difference is that biocatalysts replace expensive metal catalysts and use organic compounds as fuel to perform oxidation reactions on the anode and release electrons [30]. Taking the glucose/O_2_ biofuel cell as an example, the overall and electrode reactions are as follows:

(1) $$Anode:Glucose\overset{{GO}_{X}}{\to }Gluconolactone+2H^{+}+2e^{-}$$

(2) $$Cathode:4H^{+}+4e^{-}\overset{Lac}{\to }2H_{2}O$$

(3) $$Total:Glucose+O_{2}\overset{Enzyme}{\to }Gluconolactone+2H_{2}O$$

Compared with traditional fuel cells, biofuel cells reduce steric hindrance, increase the volume activity of the catalyst, and facilitate the transportation of fuel directly to the catalyst [32]. The entire biofuel cell constitutes a system that is capable of converting chemical energy into electrical energy. Compared with methanol, borohydride, etc., which are used in traditional fuel cells, biofuel cells use various types of organic matter (such as wastewater, body fluids, etc.) as fuel, which is safer, renewable, and easier to handle [33].

In a biofuel cell, the bioanode and biocathode are partitioned by a polymer membrane. This membrane plays a crucial role in regulating the ion transfer between the bioanode and biocathode, while also preventing the diffusion of reaction byproducts into the opposite compartment of the cell. Nonetheless, the incorporation of such membranes typically leads to increased internal cell resistance, which in turn can result in a reduction in the power output. This is a key consideration in the design and efficiency of biofuel cells. Due to the specific selectivity of enzyme-catalyzed reactions, the need for membrane separators is eliminated [34,35].

In cell assembly, the anode usually uses oxidase or dehydrogenase to catalyze the oxidation of biomass fuel, such as glucose oxidase/dehydrogenase (GOD/GDH), lactate oxidase/dehydrogenase (LOD/LDH), alcohol oxidase/dehydrogenase (AOD/ADH), formaldehyde dehydrogenase, formate dehydrogenase, etc. The cathode uses multi-copper oxidase (MCO), cytochrome oxidase (COx), or peroxidase (HRP) to catalyze the reduction of oxygen or hydrogen peroxide to achieve efficient conversion of biomass chemical energy into electrical energy [36,37]. Compared with precious metal catalysts, there are many kinds of biological enzymes, with high catalytic activity and selectivity and mild catalytic conditions. These characteristics make enzymatic biofuel cells not only have the advantages of general fuel cells, but also have the advantages of wide fuel sources, mild operating conditions, high equipment safety, good biocompatibility, and a low cost [38].

### 2.2. Classification and Characteristics of Enzyme Biofuel Cells

In enzymatic biofuel cells, the process of electron transport is critical. However, due to the intricate structure of biocatalysts, efficiently transporting electrons that are generated at the reaction site to the anode presents a significant challenge. One of the key issues is that bioactive catalysts positioned on electrode surfaces typically do not facilitate substantial direct electron transfer communication with conductive supports and redox centers. This limitation primarily arises because the biocatalytic site is often shielded by the surrounding protein matrix, which hinders direct electron transfer to the electrode. Therefore, direct electron transfer requires special biocatalysts and conditions, and most enzymatic biofuel cells require additional electron mediators as intermediaries for electron transfer [24,39].

Overall, electron transfer between active sites and immobilized materials in enzymatic biofuel cells can be categorized into two types: indirect electron transfer (MET) and direct electron transfer (DET). In the MET approach, certain biocatalysts are devoid of electrochemically active proteins on their surfaces, necessitating the use of a mediator to facilitate electron transfer from the biocatalyst to the anode. These electroactive mediators can be further classified into synthetic and natural types. The mediator, characterized by its reversible electrochemical properties, acts as a redox agent that assists in transferring electrons between the oxidoreductase’s coenzyme/cofactor and the electrode. For instance, when using glucose oxidase (GOx) in biofuel cells, the presence of an insulating protein shell around GOx makes it imperative to employ a mediator for the conveyance of electrons to the anode. Electronic mediators are usually divided into two categories: metal-based media and oxazine-based media [40,41].

In DET, some biocatalysts are electrochemically active and can transfer electrons to the electrode surface without the need for a medium. This type of electron transfer process is called DET. Enzymes that are capable of DET typically have relatively exposed active sites or small tunneling distances of approximately 1.5 nm. However, only a few biocatalysts can transfer electrons directly to the anode, and these catalysts are very sensitive to pH, temperature, and electrolyte concentration [42,43]. However, DET has some advantages over MET, including not needing membranes and compartments, a simpler design, and a smaller cell size.

### 2.3. Commonly Used Enzymes in Enzyme Biofuel Cells

Glucose oxidase (GOx, EC1.1.3.4) is the most commonly used enzyme in bioanodes. It is easily obtained from Aspergillus niger and is widely used in the development of and research on bioanodes. Compared with other oxidases, it has higher activity, specificity, and stability in the conversion of β-D-glucose. Wilson and Turner et al. [44] believed that it was an ideal enzyme that could be applied to glucose sensors in the early 1990s. GOx has the coenzyme factor FAD, and its redox potential is about −0.45V (vs.Ag/AgCl). Structurally, GOx is an enzyme with large molecular size and high molecular weight, and it exhibits stable catalytic activity [45]. This is because the FAD cofactor that is buried deep within the protein shell is not easily inactivated. However, due to the deep location of the active site of the enzyme and the long tunneling time due to the macromolecular structure of the enzyme, it is difficult to transfer electrons on the electrode surface [46]. After material modification on the electrode surface, DET can be achieved through electron tunneling between the enzyme and the electrode. A medium with an appropriate redox potential can also be used to transfer electrons from the catalytic site to the electrode surface, i.e., MET. Zebda et al. [47] used GO_x_ electrodes modified and functionalized with carbon nanotubes, and the GBFC without electronic mediators can provide a high power density of approximately 1.3 mWcm^−2^ and an open circuit voltage of 0.95 V.

The biocathode of an EBFC mainly uses enzymes of the multi-copper oxidase family, of which the more representative ones are laccase (Lac, EC1.10.3.2) and bilirubin oxidase (BOD, EC1.3.3.5) [48]. They can reduce oxygen to water without the production or involvement of hydrogen peroxide (H_2_O_2_). These enzymes consist of four copper atoms constituting the catalytic active center and are divided into three types. T1 is mainly used for substrate oxidation, and T2/T3 are three-atom copper clusters, which are beneficial to the reduction of O_2_ [49].

Since the T1 site is positioned near the protein surface, employing an auxiliary enzyme orientation method can aid in minimizing the distance of the electron channel between the electrode surface and the T1 site. Laccase, known for its relatively high redox potential ranging from 400 mV to 800 mV versus NHE, is extensively utilized as a green and recyclable energy source. The electron transfer reactions in laccase primarily fall into two categories. In the case of MET (Mediated Electron Transfer), the design structure of the electrode–enzyme interface is intricate, leading to a high overpotential in the electrocatalytic process and consequently a diminishing operational stability. However, this issue is not prevalent in the DET (direct electron transfer) reaction. For DET, it is crucial that the redox site of the enzyme, namely, the T1 site, is oriented towards the electrode to facilitate effective electron transfer. And when the enzyme is immobilized on the electrode, the distance between the redox site and the electrode must be the shortest [50].

Enzyme cascade oxidation is a strategy that utilizes the synergistic effect of multiple enzymes to achieve deep or complete oxidation of fuel, thereby improving the energy density and power density of enzymatic biofuel cells. Enzyme cascade oxidation can be divided into two methods: in vivo and in vitro [51]. The former uses the enzyme system in the organism, such as the glycolysis pathway, citric acid cycle, etc., and the latter uses an artificially designed multi-enzyme reaction system. Research on enzymatic cascade oxidation of disaccharides or complex sugars mainly focuses on glucose, fructose, xylose, cellulose, starch, etc. Currently, a variety of enzyme electrodes and enzyme reactors have been developed to achieve efficient utilization of these fuels [52].

### 2.4. Immobilization of Enzymes

**Figure 2:**
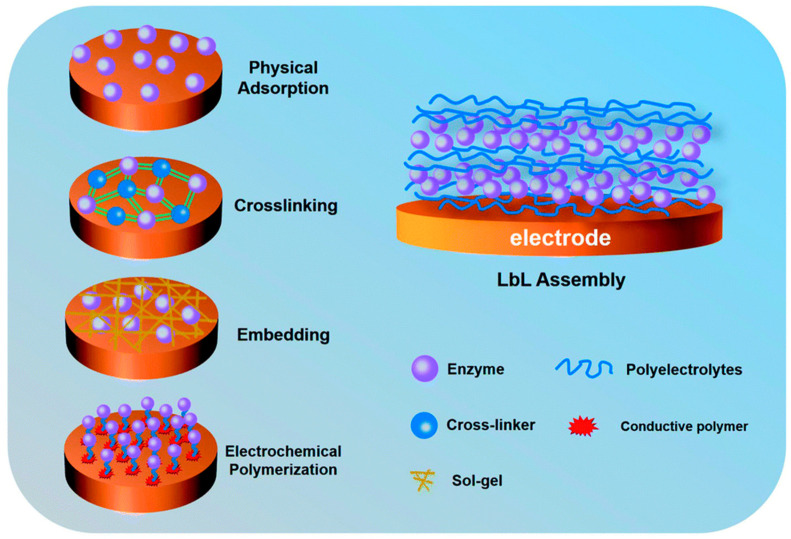
Enzyme immobilization strategy in enzymatic biofuel cells. Reprinted/adapted with permission from Ref. [53]. 2020, Royal Society of Chemistry.

Oxidoreductases are usually composed of an apoenzyme (the protein part of the enzyme structure) and a coenzyme (a small, non-protein, electrically active substance) [53]. The presence of coenzymes ensures electron transfer between the enzyme and the substrate, as shown in Figure 2. Coenzymes may be tightly fixed to the enzyme structure during the reaction, or they may be released from the active site of the enzyme. Coenzymes that are commonly used for glucose oxidation include flavin adenine dinucleotide (FAD), nicotinamide adenine dinucleotide (NAD), and pyrroloquinoline quinone (PQQ).

Some enzymes have tightly connected coenzymes at their active sites, allowing electrons to be transferred directly from the enzyme to the electrode, a process called direct electron transfer (DET). Although direct electron transfer has been realized in many enzymes [54,55,56], there are still many difficulties that need to be overcome in order to increase the DET rate and achieve sufficient current density at the active site and electrode surface. For example, the electron transfer rate has an exponential relationship with the distance between the electron donor and the acceptor, and the rate is negligible when the distance between the two exceeds 2 nm [57]. This implies that direct electron transfer (DET) can only occur when the distance between the cofactor and the active site, relative to the electrode, is within a span of 2 nm. Additionally, it is imperative for each enzyme to be correctly oriented on the electrode surface. This proper orientation is crucial to ensure that the active site is at the closest possible distance to the electrode. Lastly, it is important to note that even if the aforementioned conditions are satisfied, the generation of a bioelectrocatalytic current is not guaranteed. This is because the electrodes themselves might impede the access of the substrate to the enzyme’s active site, thus hindering the bioelectrocatalytic process.

As an alternative to direct electron transfer (DET), the process of Mediated Electron Transfer (MET) utilizes small artificial substrates or cosubstrates, alongside electroactive molecules known as mediators, to facilitate electron transfer between enzymes and electrodes. The thermodynamic redox potential of the electron mediator in MET is critical, as it defines the maximal open-circuit voltage of the enzymatic biofuel cell. For efficient electron transfer in MET, it is essential that the redox potential at the anode be more negative, and at the cathode more positive. This arrangement enhances electron transfer between the mediator and the enzyme’s active site, but it also results in voltage loss within the cell, which is a crucial factor to consider for the overall cell efficiency [58,59]. Consequently, the primary challenge encountered in Mediated Electron Transfer (MET) systems is attaining an equilibrium between the driving force and current to optimize power output. Initial investigations into MET concentrated on incorporating mediators into the liquid phase. This necessitates the use of a separator between the two poles of the cell to prevent the mediators from disrupting the reaction occurring between them. Therefore, immobilizing both the mediator and the enzyme on the electrode surface can aid in the miniaturization of enzyme biofuel cells. It also paves the way for constructing enzyme biofuel cells without the need for a separator, streamlining the design and potentially enhancing efficiency.

Since the electrode materials of supercapacitors have a high specific surface area, high conductivity, and high capacitance, the electron transfer and charge storage between the enzyme and the electrode can be enhanced. The energy density and power density of enzymatic biofuel cells can be improved. Through the good chemical stability and cycle stability of the electrode material of the supercapacitor, the enzyme can be protected from denaturation and deactivation, and the life of the enzyme can be extended [60].

However, the inherent fragile properties and low tolerance of free enzymes, such as sensitivity to temperature and pH changes, limit the wide application of enzyme catalysts [61]. Furthermore, the enzymes themselves are sources of contamination, leading to unavoidable purification and isolation steps [62]. Therefore, immobilized enzyme technology was proposed and extensively studied [63]. Immobilized enzyme technology is a technology that limits enzyme catalysts to a specific spatial range through certain physical and chemical effects, which can effectively improve the stability of enzyme catalysts [64]. Furthermore, the heterogeneous nature of immobilized enzymes provides a more convenient separation method to reduce secondary contamination. Not only that, the recyclable nature of the carrier facilitates the reuse of enzyme catalysts, improving their cost-effectiveness [65]. More importantly, the properties of enzyme catalysts are closely related to the properties of the carrier and have excellent designability [66]. Current strategies for combining enzymes with electrodes mainly include four methods: adsorption, covalent binding, cross-linking, and trapping [67].

## 3. The Application of Nanomaterials in Enzyme Biofuel Cells

The bioelectrocatalytic efficacy of immobilized enzymes is significantly influenced by the conductivity between the enzyme’s redox center and the electrode. Enhancing the surface properties of the electrode and refining the method of enzyme immobilization can lead to improved electron transfer efficiency at the electrode. It is crucial to ensure efficient electron transfer between the redox center of the immobilized enzyme and the surface of the electrode material. Consequently, when selecting materials for immobilization, their conductivity and stability are primary factors to consider.

With advancements in materials science, a variety of materials, such as polymers, carbon materials, metal nanomaterials and their oxides, sol–gel techniques, and various composite materials, have emerged as focal points of research. These materials are increasingly being utilized as efficient immobilization substrates in enzymatic biofuel cells (EBFCs), as illustrated in Table 2. The incorporation of these materials has had a profound impact on the evolution and advancement of EBFC technology.

It should be noted that nanomaterials may also have some negative effects on enzymes:

**Oxidative stress**: Nanomaterials can induce the production of reactive oxygen species (ROS) that damage membranes, DNA, and proteins of enzyme-producing cells. ROS can also inhibit the activity and stability of enzymes by changing their structure and function.

**Inhibiting enzyme activity**: Nanomaterials can interact with the active site or cofactors of enzymes, reducing their catalytic efficiency. Nanomaterials can also prevent substrate access to enzymes or interfere with the formation of enzyme–substrate complexes.

**Alterations in enzyme expression**: Nanomaterials can affect the gene expression and regulation of enzymes by modulating transcription factors, signaling pathways, or epigenetic mechanisms. Nanomaterials can also cause DNA damage or mutations that impair enzyme synthesis and function.

Therefore, the above effects need to be considered when designing and optimizing nanomaterials for use in enzymatic biofuel cells.

In the past few decades, the use of nanomaterials has become an effective means to improve biofuel cells’ processing efficiency [68,69], and researchers have developed a variety of nanomaterials for EBFCs [70,71,72]. Nanomaterials have unique properties such as a high surface area, strong adsorption capacity, and easy storage. Some nanomaterials themselves have a certain catalytic activity and can be recycled repeatedly. Their stability, durability, and reusability are very good, helping optimize the entire fuel cell system [73]. Therefore, various carbon-based nanomaterials (CBNMs), metal nanoparticles, and inorganic nanoparticles have been extensively studied by scientists to improve the performance of biofuel cells.

### 3.1. Carbon Nanomaterials and Cell Performance

Carbon-based materials are widely used as electrode materials for EBFCs. They have superior conductivity, a large surface area, high porosity, and good thermal and chemical stability. As the research on nanomaterials continues to deepen, various carbon-based nanomaterials, such as carbon nanotubes (CNTs), porous carbon, and conductive graphene, are used to improve the efficiency of EBFCs [25,74]. EBFCs using carbon-based materials have increased conductivity and a reduced size and weight of electrodes, and they are widely used in implantable or wearable power sources [24]. Despite their excellent properties, the biosafety of various conductive carbon materials for long-term use in implantable devices remains controversial due to their cytotoxicity issues [75,76].

**Figure 3:**
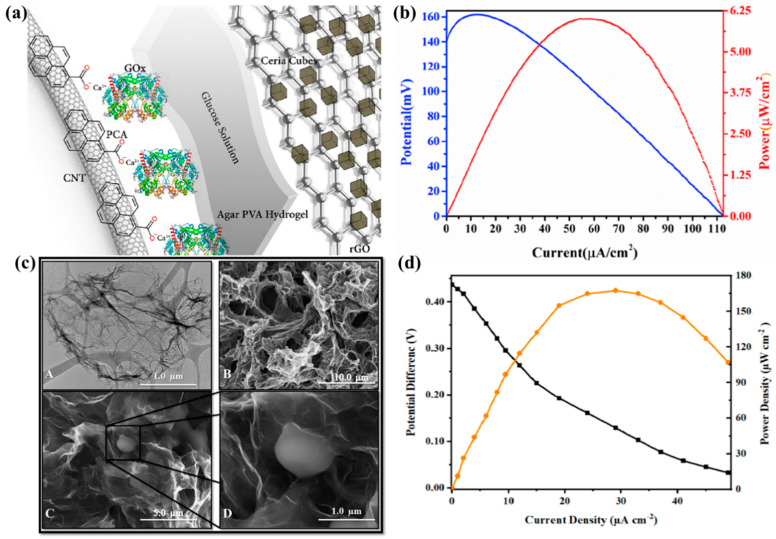
(**a**) Carbon nanotube-immobilized enzyme as anode; (**b**) Electrochemical performance of sandwich-structured glucose enzyme fuel cell. Reprinted/adapted with permission from Ref. [77]. 2019, Elsevier; (**c**) SEM image of 3D graphene structure; (**d**) Electrochemical performance diagram of 3D graphene as EBFC electrode. Reprinted/adapted with permission from Ref. [82]. 2019, Elsevier.

Arjun et al. [77] used a hierarchical structure composed of CNTs that were acidified with 1-pyrenecarboxylic acid (PCA) and calcium ions to immobilize glucose oxidase (GOx) through electrostatic adsorption as an enzyme anode, as shown in Figure 3a. Compared with CNTs that are acidified with PCA and CNTs that are acidified with nitric acid/sulfuric acid, the surface morphology of the obtained CNTs is not destroyed, and the current density of the fixed GOx anode is higher. A sandwich-structured glucose enzyme fuel cell was constructed using reduced graphene oxide ceria as a non-enzyme cathode. The recorded open circuit voltage was 140 mV at 60 μA/cm^2^, and the peak power density was 6.25 μW/cm^2^, as shown in Figure 3b. When this EBFC is used to charge a capacitor, the capacitor can be charged to approximately 400 mV. This shows that the EBFC has the ability to serve as an independent power source.

Graphene has excellent electrical conductivity and a very high specific surface area and mechanical strength [78], so it has become the main nanomaterial for constructing enzymatic biofuel cells. Liu et al. [79] used graphene to construct a bioanode in an EBFC for the first time. Glucose oxidase, graphene, and redox mediators were sol–gel embedded in tetramethoxysilane on a gold substrate. The preparation method of biocathode is the same as that of bioanode, and the cathode enzyme is bilirubin oxidase. The maximum power density of the cell that was constructed in this way is (24.3 ± 4) μW. After 7 days, the power output dropped to 50% of its original power output. For comparison, a CNT was used instead of graphene, and the enzyme anode was prepared using the same method and assembled into an EBFC. After analysis, the EBFC based on a graphene anode has a higher power density.

Some researchers have used 3D graphene to improve the performance of EBFCs [80,81]. Babadi et al. [82] constructed an EBFC based on 3D graphene through the Hummers method and hydrothermal method and then modified it with glucose oxidase on the glassy carbon electrode, as shown in Figure 3c. The bioanode has a power density of 164 μW/cm^2^ at 0.4 V. The highly porous structure of 3D graphene loads more GOx and reduces enzyme leaching, as shown in Figure 3d. The enzyme loading capacity and electrode stability are improved, and the electron transfer between the GOx active site and GCE is promoted. This significantly improves the electron transfer rate between the enzyme and the electrode, promotes the immobilization of the enzyme on the electrode surface, and extends the life of the enzyme. Compared with other glucose biofuel cells, the open circuit voltage of this enzyme biofuel cell reaches 0.4V, and the maximum output power density reaches 164 μW/cm^2^.

### 3.2. Metal Nanoparticles and Cell Performance

**Figure 4:**
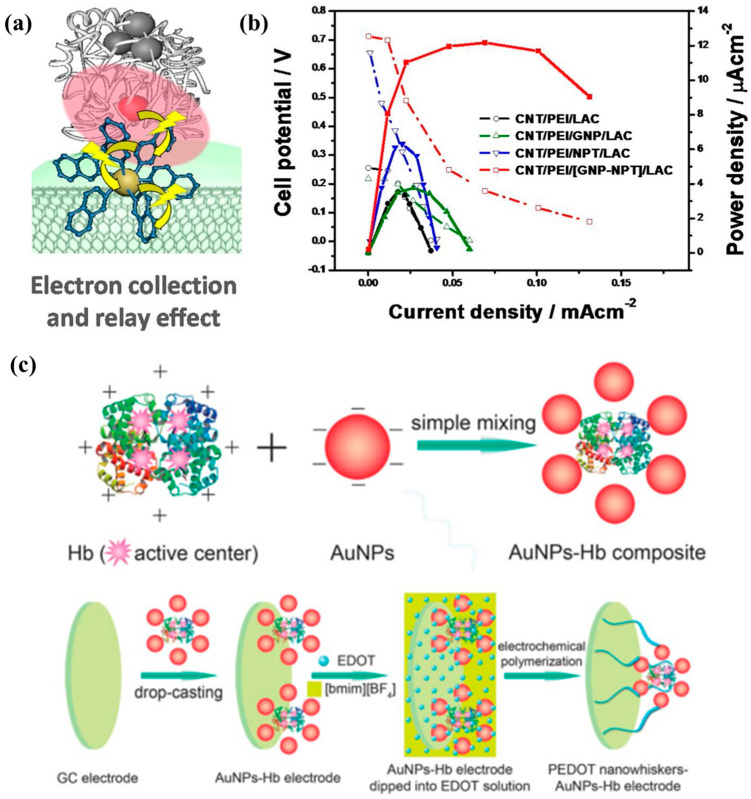
(**a**) AuNPs immobilized enzyme as anode and its electrochemical performance (**b**) Reprinted/adapted with permission from Ref. [89]. 2018, Elsevier; (**c**) Schematic diagram of AuNPs promoting ethanol dehydrogenation. Reprinted/adapted with permission from Ref. [90]. 2013, Royal Society of Chemistry.

Oxide and metal nanomaterials have been extensively studied in biofuel cell design due to their unique physical and chemical properties. For example, Au, Ag, and Pt nanoparticles and some functional oxide nanoparticles (such as Fe_2_O_3_, Al_2_O_3_, ZnO, and TiO_2_) are used in biofuel cells or biocatalysis due to their high thermal stability, excellent electron transfer performance, and high catalytic performance [83,84,85]. Various metal nanoparticles such as AuNPs, AgNPs, PtNPs, etc., have been used to modify the electrodes of EBFCs to promote electron transfer between the enzyme and the substrate electrode [50,86]. In some cases, AuNPs are modified on surfaces such as carbon nanotubes, graphene, or conductive polymers. The enzyme is immobilized on the substrate electrode through the formation of a chemical bond through a chemical reaction between the end group of the enzyme and the groups on the pretreated AuNPs [87,88]. Electron transfer is facilitated by reducing the distance between the enzyme and the substrate electrode. Suhyeon et al. [89] artificially improved the performance of a laccase-based cathode EBFC and prepared an electrode composed of AuNPs wrapped with laccase, CNTs, polyethylenimine (PEI), and 2-naphthalenethiol (NPT). Among them, PEI immobilizes laccase through electrostatic adsorption. Pt/C was used as the anode, and glucose aqueous solution was used as the electrolyte (40 mmol/L), as shown in Figure 4a. The maximum power density of this EBFC is 13 μW/cm^2^, which is significantly better than the electrode without AuNPs (7 μW/cm^2^), as shown in Figure 4b. This shows that the gold–sulfhydryl bond can promote electron transfer between the CNT/PEI substrate and laccase, greatly improving the oxygen reduction rate and EBFC power density of the laccase cathode.

In order to realize direct electron transfer on the bioanode of alcohol dehydrogenase, Ratautas et al. [90] prepared a bioanode composed of alcohol dehydrogenase, AuNPs, and 4-aminothiophenol. AuNPs are prepared by reducing HAuCl_4_ with sodium citrate and then drop-coated onto a gold-based electrode. 4-aminothiophenol modifies alcohol dehydrogenase on the AuNPs through gold–sulfur bonds and covalent bonds, as shown in Figure 4c. The electrode was used to catalyze glycerol, and electrochemical tests were performed, showing that the bioanode produced a current density of 510 μA/cm^2^ at pH = 7.0 and 0 V/SCE. This is mainly because AuNPs promote electron transfer between the redox center of alcohol dehydrogenase and 4-aminothiophenol-modified AuNPs.

### 3.3. Conductive Hybrid Materials and Cell Performance

Carbon nanomaterials are employed to enhance the electrode’s surface area and its conductivity. Metal nanoparticles, on the other hand, serve to reduce the electron transfer distance between the enzyme and the base electrode. This facilitation of electron transfer can lead to an increase in both the electrode current density and the power density of enzymatic biofuel cells (EBFCs). By hybridizing these two types of materials, the overall performance of enzyme electrodes and EBFCs can be significantly improved. This hybrid approach synergistically combines the beneficial properties of both carbon nanomaterials and metal nanoparticles, thereby optimizing the efficiency of the bioelectrocatalytic process.

**Figure 5:**
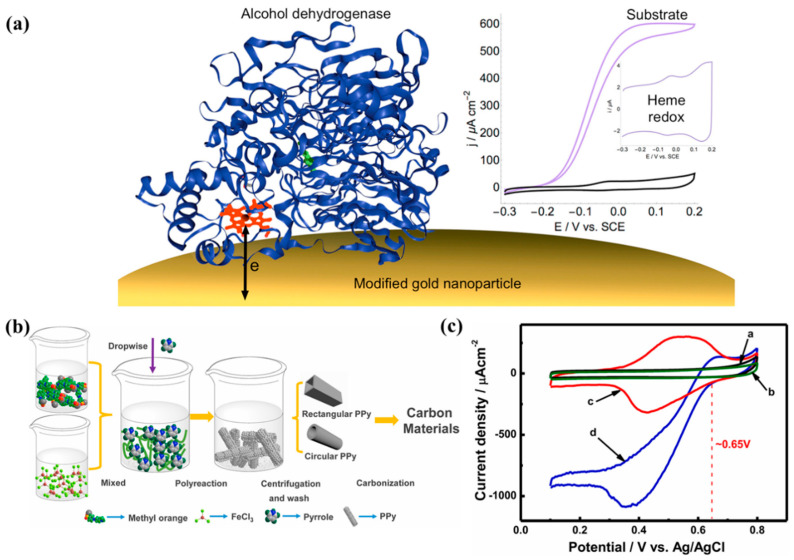
(**a**) Preparation of N-CNTs in 3D-RGO/Au and its electrochemical properties. Reprinted/adapted with permission from Ref. [91]. 2017, Elsevier; (**b**); Enzyme fuel cell of carbonized RPPy-based GOx and its electrochemical properties (**c**) Reprinted/adapted with permission from Ref. [92]. 2019, Elsevier.

Zhang et al. [91] used electrodeposition and chemical vapor deposition (CVD) methods to prepare a novel three-dimensional electrode structure. The researchers employed a method where they electrodeposited reduced graphene oxide (RGO) onto the surface of nickel foam, thereby creating a three-dimensional RGO network. Within this structure, Au (gold) nanoparticles were codeposited. Following this, a straightforward chemical vapor deposition (CVD) process was used to seamlessly grow nitrogen-doped carbon nanotubes (N-CNTs) on the surface of the three-dimensional RGO/Au nanoparticles. The presence of Au nanoparticles and the nitrogen doping within this nanostructured composite are crucial, as they provide a significantly higher number of active sites for bioelectrocatalysis. This innovative approach effectively combines the conductive and structural benefits of RGO and N-CNTs with the catalytic properties of Au nanoparticles, thereby enhancing the overall bioelectrocatalytic performance. In addition, N-CNTs can provide a high specific surface area for enzyme immobilization, promote electron transfer between glucose oxidase and electrodes, and exhibit a current density as high as 7.02 mA/cm^2^ (0.3V vs.Ag/AgCl). The glucose/air biofuel cell combined with a platinum cathode has an open circuit voltage of 0.32 V and produces a maximum power density of 235 μW/cm^2^ at 0.15V, as shown in Figure 5a. There is a synergistic effect between N-CNT, RGO, and Au nanoparticles in this three-dimensional structure, giving it a higher specific surface area, enzyme loading capacity, and more bioelectrocatalytic active sites. It shows that the use of conductive hybrid materials to construct three-dimensional bioelectrodes can help solve the problems of the low output power and low current density of EBFCs.

Kang et al. [92] prepared a new type of carbon tube by carbonizing rectangular polypyrrole tubes at a high temperature for constructing high-performance enzymatic biofuel cells. GOx or Lac electrodes based on carbonized RPPy modification exhibit excellent bioelectrochemical properties, as shown in Figure 5b. The open circuit voltage of the biofuel cell (GOx, glucose/O_2_, Lac) that they assembled reached 1.16 V, and the maximum power density was 0.350 mW/cm^2^, as shown in Figure 5c. Kim et al. [93] immobilized GOx into the porous matrix of polyaniline nanofibers through a three-step process, including enzyme adsorption (EA), precipitation, and cross-linking (EAPC). After EA and EAPC treatment, the maximum power densities of EBFCs built based on these bioanodes were 57 and 292 μW/cm^2^, respectively. After heat treatment at 60 °C for 4 h, the maximum power densities of the EA and EAPC anodes were 32 and 315 μW/cm^2^, respectively, accounting for 56% and 108% of the maximum power density that was initially obtained.

These analyses reveal that such nanomaterials are capable of reducing the electron transfer distance between the substrate electrode and the enzyme, while also enabling the loading of a greater quantity of enzymes. This is attributed to their excellent conductivity and expansive specific surface area. Consequently, the utilization of conductive nanomaterials, including carbon materials, metal nanoparticles, or hybrid conductive nanoparticles, for constructing three-dimensional bioelectrodes can significantly increase the specific surface area of enzyme electrodes. This enhancement leads to a faster electron transfer rate, improved performance of enzyme electrodes, and an increase in both the current density and output power of enzymatic biofuel cells (EBFCs). The strategic employment of these nanomaterials thus plays a pivotal role in optimizing the efficiency and functionality of EBFCs.

**Table 2:** Nanomaterials applied in enzymatic fuel cells.

Nanomaterials	Performance	Application
Graphene/carbon nanotube/enzyme composite fabric [94]	High conductivity, high specific surface area, high enzyme loading, high catalytic efficiency, high stability, high stretchability	Flexible biofuel cells for biosensing and bioimaging
Open silicone nanocapsules [95]	High enzyme loading, high catalytic efficiency, high stability, high reaction rate, high selectivity	Enzyme fuel cell for catalyzing methanol oxidation
Single atom nanozyme [96]	High atomic utilization, high active center density, high catalytic activity, high stability, high selectivity	Enzyme fuel cell can be used to catalyze glucose oxidation, hydrogen oxidation, oxygen reduction, etc.
Thermoplastic polyurethane rubber/carbon nanotube/enzyme composite fiber [97]	High biocompatibility, high enzyme loading, high catalytic efficiency, high stability, high stretchability	Implantable biofuel cell for catalyzing glucose in body fluids

## 4. Self-Powered Sensors Based on EBFCs

The initial motivation for EBFC research was to recycle human metabolites as green energy or as energy supply equipment for implantable medical devices [98,99]. However, as EBFC performance (such as stability and power output) reached a bottleneck, researchers began to introduce EBFCs into the field of biosensing and develop EBFC-based self-powered electrochemical biosensors (EBFC-SPBs) [100,101].

Compared with traditional electrochemical biosensors, an EBFC-SPB has its unique features [102,103]. First, the detection limit of an EBFC-SPB is generally low, making it a promising ultrasensitive trace detection tool. An EBFC-SPB is a dual-electrode system that does not require a reference electrode and is easier to miniaturize. In addition, the self-powering capability of an EBFC-SPB allows the device to be used in the field of portable online monitoring. The working principle of an EBFC-SPB is that the power output of the EBFC is closely related to the concentration of analytes, and the power output of the EBFC is also directly related to the enzyme loading on the anode/cathode [71,104,105]. According to the working principle of EBFC-SPBs, the design ideas of traditional self-powered sensors are usually based on the following aspects: substrate effect [106,107], inhibition effect [108,109], enzyme effect [40,109], steric hindrance effect [110,111], etc.

### 4.1. Substrate Effect

**Figure 6:**
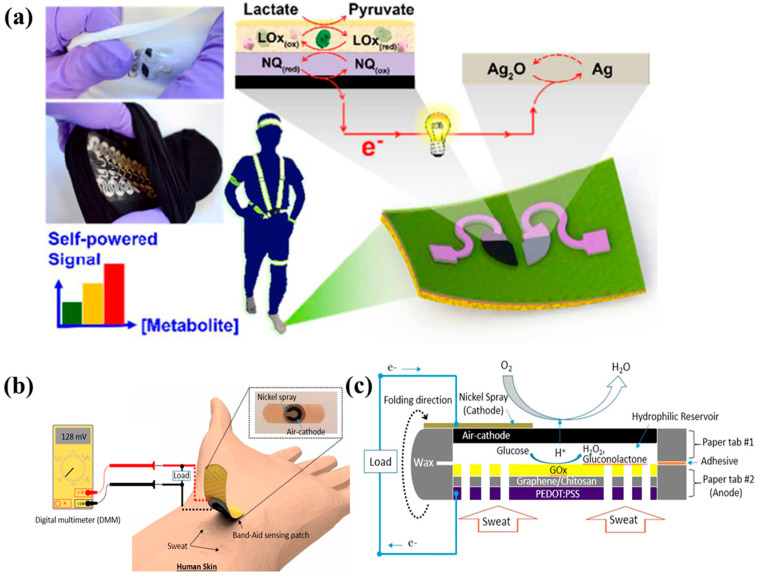
(**a**) Stretchable flexible EBFC array. Reprinted/adapted with permission from Ref. [113]. 2016, MDPI; (**b**) Non-invasive sensor for monitoring human sweat glucose concentration; (**c**) Self-supplying glucose EBFC based on screen printing technology. Reprinted/adapted with permission from Ref. [115]. 2018, American Chemical Society.

During exercise, lactate is produced when endogenous glycogen in muscle tissue is broken down into pyruvate via the glycolytic pathway, which is subsequently reduced by lactate dehydrogenase [112]. Throughout the process, the rate of lactate production far exceeds the rate of consumption, so the lactate concentration increases in proportion to the degree of physical exertion. Lactate then circulates throughout the body via monocarboxylic acid transporters and can be detected in sweat, tears, urine, saliva, and serum at typical concentrations of 20 mM, 3 mM, 0.1 mM, 0.5 mM, and 1mM, respectively. Wang et al. [113] proposed the first stretchable flexible EBFC array, which can harvest energy from human sweat and can also be used as a non-invasive, wearable device for self-powered quantitative analysis of lactic acid content in the body, as shown in Figure 6a. By depositing Ag_2_O/Ag on the surface of flexible textiles as a biocathode and modifying 1,4-naphthoquinone (NQ), CNTs, and lactate oxidase (LOx) on the flexible-base electrode as a bioanode, a wearable lactic acid self-powered sensor was constructed. Volunteers wear sensors on socks to monitor changes in sweat lactic acid content during exercise in real time. This EBFC-SPB array, built based on inkjet printing technology and snake-shaped design, is expected to promote the development of wearable self-powered sensors.

The glucose concentration in the human body is closely related to human health. Choi et al. [114] developed a low-cost self-powered glucose biosensor (approximately USD 0.15) based on a glucose/O_2_ fuel cell. Based on one-dimensional origami technology, the researchers developed a paper-type self-powered glucose sensor using chitosan/glucose oxidase (GOx) as the bioanode to catalyze glucose oxidation and Ni/activated carbon as the cathode to catalyze O_2_ reduction. The detection limit of this biosensor is as low as 0.02 mA/Mm^−1^. This kind of biosensor has the advantages of low cost, no external power supply, no need for complex converters, accurate and efficient monitoring, and being potentially suitable for clinical diagnosis and environmental monitoring in developing countries. Subsequently, Choi designed the sensor on a band-aid patch based on screen printing technology and designed a non-invasive sensor to monitor the concentration of glucose in human sweat [115], as shown in Figure 6b. The sensor has ultrahigh detection sensitivity, with a detection limit of 1.35mA/Mm^−1^, which is nearly 70 times the detection sensitivity of previously reported sensors. The sensor can achieve real-time and accurate monitoring of human sweat glucose. This band-aid-type self-powered glucose biosensor has high application prospects in monitoring and managing diabetes, and the EBFC-SPB has a highly integrated simplified reading system that enables continuous non-invasive glucose monitoring, as shown in Figure 6c.

Abnormally expressed miRNAs in body fluids are considered endogenous biomarkers for cancer diagnosis. By combining EBFCs with homogeneous electrochemical technology, Li et al. [116] designed an ultrasensitive self-powered biosensor for miRNA. The cathode uses [Fe(CN)_6_]^3−^ as the reactant, and the anode uses glucose oxidase to catalyze glucose oxidation. The authors synthesized positively charged mesoporous silica nanoparticles (PMSNs) to encapsulate the reactant [Fe(CN)_6_]^3−^. The nanoparticles are encapsulated by DNA that is completely complementary to the target miRNA, preventing [Fe(CN)_6_]^3−^ from leaking into the electrolyte. When the miRNA-21 to be tested exists, it can specifically open the DNA chain of closed PMSNs, causing [Fe(CN)_6_]^3−^ to leak, thereby causing the EBFC’s open circuit voltage to increase. By establishing the relationship curve between the open circuit voltage of the EBFC and the concentration of the target substance, quantitative information about miRNA-21 was obtained. The detection limit of the sensor was reduced to 2.7 aM, which is much lower than other reported methods. This ultrasensitive and easy-to-operate biosensor has ideal application prospects in miRNA detection. Li et al. [117] developed a self-powered immunosensor based on the substrate effect, combined with target-induced substrate release and bioconjugation immunoassay technology, for the detection of melamine (ME). Similar to the previous sensor, glucose is encapsulated in PMSNs and sealed with AuNPs-Ab. In the presence of ME, AuNPs-Ab falls off the surface of PMSNs, and glucose is released into the electrolyte, ultimately causing the EBFC’s open circuit voltage to increase. The detection limit of ME in actual samples is as low as 2.1 pM. Therefore, the application prospects of EBFC-SPBs guided by homogeneous catalysis in the field of food safety supervision for portable and on-site detection are very promising.

### 4.2. Inhibitory Effect

The fundamental principle behind the EBFC-SPB (enzymatic biofuel cell self-powered biosensor), which is predicated on the inhibitory effect, is that the presence of an analyte can diminish the activity of the enzyme, leading to a decrease in the EBFC’s output. The variation in the EBFC’s output is directly correlated with the concentration of the analyte. Consequently, EBFC-SPBs that are developed on the basis of this inhibitory effect are predominantly employed to detect substances that pose significant risks to human health, such as heavy metal ions and drug residues.

**Figure 7:**
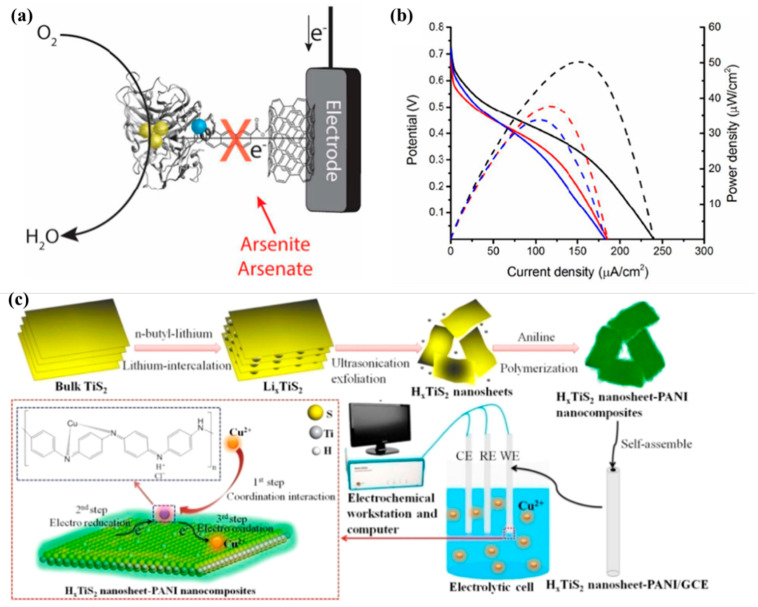
(**a**) LAC-modified biocathode and GDH (FAD-GDH)/FeMe2-LPEI as bioanode to catalyze glucose oxidation and its electrochemical performance (**b**). Reprinted/adapted with permission from Ref. [118]. 2015, American Chemical Society; (**c**) Glucose oxidase and laccase are used as biocatalysts at the anode and cathode, respectively, to construct enzymatic biofuel cells. Reprinted/adapted with permission from Ref. [119]. 2011, American Chemical Society.

Arsenic, either as a naturally occurring element or as a pollutant resulting from human activities, is a prime example of such harmful substances. Exposure to arsenic can lead to severe health issues, including skin, bladder, and lung cancers, as well as heart diseases. By utilizing EBFC-SPBs for the detection of arsenic and similar harmful substances, it becomes possible to monitor and control exposure to these hazardous elements, thereby safeguarding public health. By studying experimental evidence for Lac enzyme inhibition by arsenite or arsenate, Minteer et al. [118] developed a self-powered biosensor for the detection of arsenite or arsenate. The authors used an LAC-modified biocathode to catalyze O_2_ reduction and GDH (FAD-GDH)/FeMe_2_-LPEI as a bioanode to catalyze glucose oxidation, as shown in Figure 7a. Studies have confirmed that the activity of LAC, which catalyzes the oxygen reduction reaction at the cathode, is inhibited by arsenite or arsenate, and this inhibition is reversible. The effect of arsenite or arsenate on the bioanode shows that the presence of arsenite or arsenate does not affect the enzymatic activity of GOx. In the absence of arsenite or arsenate, the BFC has an OCP value of 723.3 mV. The maximum current and power density are 289.7 mA/cm^−2^ and 57.2 mA/cm^−2^, respectively, as shown in Figure 7b. When arsenite or arsenate is present, the work of BFC is medium. The output reduces the EBFC-SPB’s current/sensitive monitoring of arsenate or industrial arsenate. In the above work, changes in cell performance caused by target objects can be measured with only a multimeter, which provides a new strategy for convenient and on-site detection in the field of environmental pollution.

In addition to direct detection of enzyme inhibitors, enzymatic biofuel cell self-powered sensors have also been used to analyze masking agents of enzyme inhibitors. Zhao et al. [119] used glucose oxidase and laccase as biocatalysts at the anode and cathode, respectively, to construct an enzymatic biofuel cell and used it for self-powered detection of L-cysteine. In this work, Cu^2+^ will form a complex with the reduced coenzyme FADH2 in the active center of glucose oxidase, thereby inhibiting the activity of the enzyme and thereby obtaining lower output performance, as shown in Figure 7c. When L-cysteine is added, a large amount of Cu^2+^ will combine with L-cysteine through the action of Cu-S bonds, that is, the enzyme will be reactivated. The output performance of the cell is restored, and the degree of performance recovery is dependent on the concentration of L-cysteine. Similar principles were also used to develop EDTA’s enzymatic biofuel cell self-powered sensor [120].

Diverging from the detection methods that focus on enzyme inhibitors, these two sensors, designed for detecting masking agents, operate on a “signal-on” mode. This approach effectively circumvents the occurrence of false-positive signals, thereby providing a more accurate reflection of the actual concentration changes in the substance being tested. Additionally, it is common to find numerous types of masking agents corresponding to the same enzyme inhibitor. Through thoughtful and strategic design, the detectable range of self-powered sensors can be significantly expanded. This expansion in range allows for a broader spectrum of analytes to be accurately and reliably detected, enhancing the versatility and utility of these sensors in various applications.

### 4.3. Enzyme Effect

The term “enzyme effect” refers to how variations in the loading amounts of enzymes on electrodes can lead to corresponding increases or decreases in the output of enzymatic biofuel cells (EBFCs). Alterations in the amount of enzyme that is present on the electrodes of enzymatic biofuel cells are bound to impact the cell’s performance. Through thoughtful and precise design, the concentration of the analyte can be made to depend on the enzyme loading on the electrode. Consequently, changes in the cell’s performance can be observed, which then serve as an indicator of the concentration of the target analyte. This approach effectively leverages the enzyme loading as a key variable in detecting and quantifying specific substances, making the biofuel cell a sensitive tool for analytical purposes.

**Figure 8:**
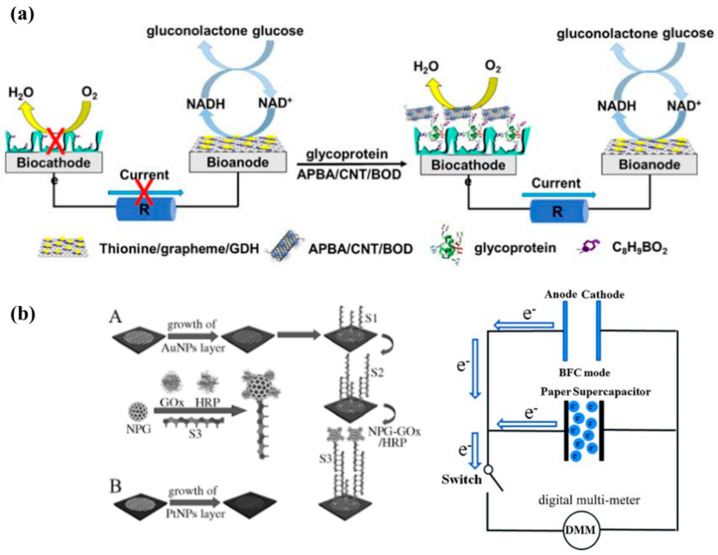
(**a**) Miniaturized self-powered biosensor based on enzyme effect on plastic substrate for monitoring ovarian cancer markers. Reprinted/adapted with permission from Ref. [121], 2014, American Chemical Society; (**b**) Glucose oxidase is immobilized on the anode based on DNA double-strand complementation technology. Reprinted/adapted with permission from Ref. [123]. 2022, American Chemical Society.

Liu et al. [121] developed a miniaturized self-powered biosensor on a plastic substrate based on the enzyme effect to monitor glycoprotein, a marker of ovarian cancer. This method first prepares GOx/CNTs nanocomposites that are functionalized with borate affinity molecule-imprinted polymers (MIPs). And the thionine/GN/GDH electrode is used as the bioanode to catalyze glucose oxidation, and MIP/glycoprotein/4-aminophenylboronic acid (APBA)/CNTs/BOx is used as the biocathode composite catalyst to catalyze O_2_ reduction, as shown in Figure 8a. Due to the specific recognition of borate MIP and glycoprotein, APBA/CNTs/BOx can be captured by the MIP functional layer on the surface of the biocathode and play an electrocatalytic role in oxygen reduction. The amount of glycoprotein directly determines the amount of captured APBA/CNTs/GOx, which directly determines the size of the EBFC output. The measurement result of alpha-fetoprotein (AFP) in human serum by this EBFC-SPB is ~12.8 ng/mL^−1^, which is completely consistent with the result of ELISA analysis. It is proven that the designed sensor achieves highly specific and ultrasensitive determination of glycoproteins in complex samples and has application value in clinical analysis.

Similar reports were also found in the work of Guo et al. [122]. The detection limit of this sensor is as low as 0.2 nmol/L, and the sensitivity is 50 times higher than the traditional ELISA method. DNA complementation technology is also used in the field of self-powered sensors for enzymatic biofuel cells. Yu et al. [123] modified the anode of the enzyme biofuel cell with DNA 1 that is partially complementary to the target DNA. At the same time, they loaded glucose oxidase into porous gold, and modified DNA 2 that was partially complementary to the target DNA on the surface of the porous gold through Au-S bonds, as shown in Figure 8b.When target DNA is present in the solution, glucose oxidase gets immobilized on the anode through DNA double-strand complementation technology, linking the cell’s performance improvement to the concentration of the target DNA. Additionally, this work introduces supercapacitors to store the electrical energy that is generated by enzymatic biofuel cells. The stored energy, accumulated in large amounts, can be released instantly, enabling the system to achieve a greater current output. This enhancement in current improves the sensitivity of the sensor and offers a novel method for detecting DNA mismatches. The inclusion of supercapacitors thus not only augments the energy storage capacity of the system but also paves the way for advanced DNA detection techniques.

### 4.4. Steric Hindrance Effect

**Figure 9:**
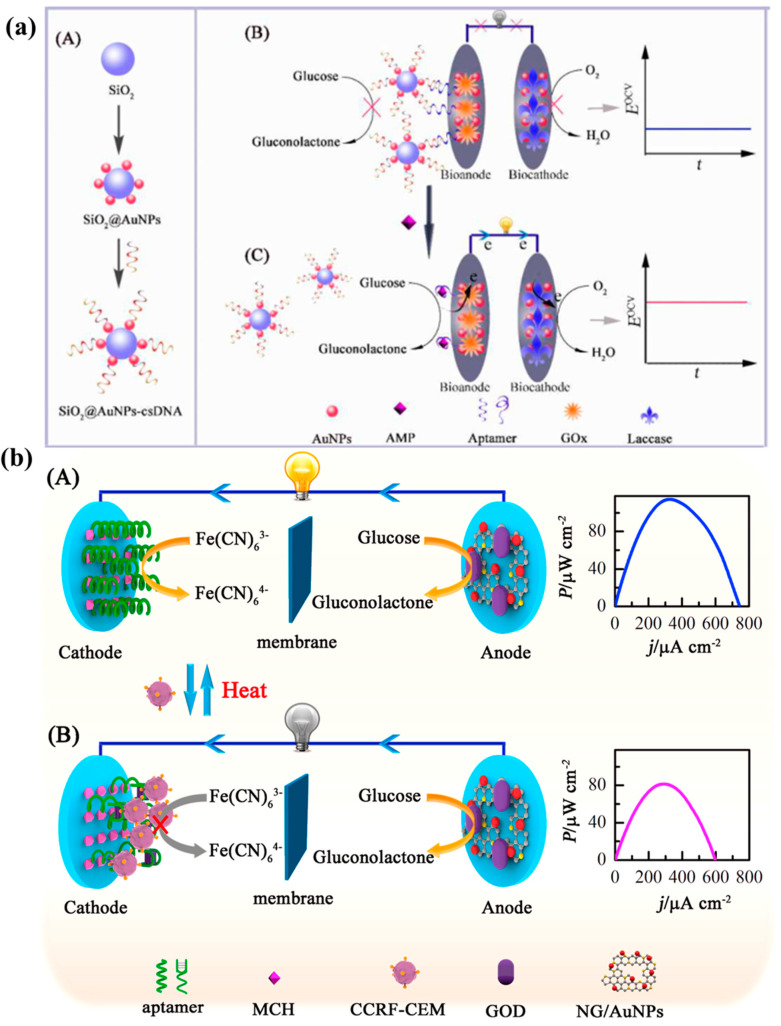
(**a**) EBFC-SPB without separator based on steric hindrance effect for monitoring antibiotic residues, Reprinted/adapted with permission from Ref. [125]. 2015, Royal Society of Chemistry; (**b**) CCRF-CEM cells based on steric hindrance effect of cathode, Reprinted/adapted with permission from Ref. [127]. 2010, American Chemical Society.

The principle of an EBFC-SPB that is prepared based on the steric effect is that the analyte is specifically enriched or detached from the electrode, causing the steric resistance on the electrode to increase or decrease, which directly leads to corresponding changes in the output of the EBFC. Quantitative information of the target is obtained based on changes in EBFC output [110,124]. Li et al. [125] proposed a separator-free EBFC-SPB for monitoring antibiotic residues based on the steric hindrance effect. The authors used AuNPs/GOx/aptamer-DNA as the bioanode and laccase (Lac)-modified electrode as the biocathode to prepare the EBFC-SPB. In the absence of AMP, DNA-chain-modified silica (SiO_2_@AuNPs-csDNA) that is complementary to the aptamer is captured by the anode, as shown in Figure 9a. Its steric hindrance effect at the anode prevents the transfer of glucose to the active site of GOx, resulting in a low open circuit voltage of the EBFC. The appearance of AMP leads to a conformational change in the aptamer, causing the SiO_2_@AuNPs-csDNA to fall off the bioanode, reducing the steric hindrance effect and increasing the EBFC open circuit voltage. The EBFC-SPB can detect AMP in milk samples with a detection limit of 3 pM, which is better than the sensitivity of standard monitoring methods.

Based on the steric effect, Zhu et al. [126] developed a self-powered cell sensor with a unipolar chamber for specific monitoring of CCRF-CEM cells. In order to promote the electrical transfer between the enzyme and the electrode surface, the authors used a GN/CNT/AuNP hybrid as the base material of the cathode and anode. The biocathode was modified with cell aptamers to specifically capture CCRF-CEM cells, and the anode was modified with PQQ-GDH to catalyze glucose oxidation. BOx/CNTs/AuNPs serve as signal probes and are immobilized on the aptamer-functionalized GN/CNTs gold electrode through partial base pairing with the aptamer. In the absence of CCRF-CEM cells, the BOx/CNTs/AuNPs-GN/CNTs/AuNPs biocathode can efficiently catalyze oxygen reduction. The EBFC has a high open circuit voltage, and CCRF-CEM is completely complementary to the biocathode aptamer. Being captured by the biocathode causes BOx/CNTs/AuNPs to fall off the cathode, which increases the cathode steric resistance and leads to a decrease in the amount of BOx in the cathode catalyst and a decrease in the EBFC opening. The decreasing trend of the EBFC open circuit is linearly related to the amount of cells. The detection limit of the EBFC-SPB cell sensor for CCRF-CEM is as low as five cells.

Later, Zhang et al. [127] also used the steric hindrance effect to develop an ultrasensitive EBFC-SPB cell sensor for measuring CCRF-CEM cells, as shown in Figure 9b. The authors used a CCRF-CEM cell-aptamer-modified Au electrode as the cathode and [Fe(CN)_6_]^3−^ as the cathode reactant, and nitrogen-doped graphene/AuNPs/GOx (N-GN/AuNPs/GOx)-modified carbon paper electrode was used as a bioanode to catalyze glucose oxidation. Without CCRF-CEM cells, the EBFC works normally, and the power output is higher. Micron-sized CCRF-CEM cells can be specifically captured by the cathode, producing a steric hindrance effect at the cathode that significantly hinders the electron transfer between [Fe(CN)_6_]^3−^ and the cathode surface, resulting in a significant reduction in EBFC power output. The decrease in EBFC output is linearly related to the concentration of CCRF-CEM cells, and the sensor can monitor four CCRF-CEM cells. In addition, by incubating the EBFC-SPB at a high temperature after capturing CCRF-CEM cells, the cells can fall off the cathode while maintaining the stability and recognition performance of the cathode aptamer. Therefore, the EBFC-SPB can be reused, and the detection sensitivity of the reused EBFC-SPB is not significantly disturbed.

Given its ultrahigh sensitivity and reusability, this enzymatic biofuel cell self-powered biosensor (EBFC-SPB) is poised to be highly impactful in early tumor diagnosis and prognosis assessment, aiming for multi-objective analysis. Dong et al. [128] innovated a self-powered aptamer logic gate sensor based on the steric hindrance effect, constructing a separator-free microfluidic chip enzyme biofuel cell with a glucose oxidase/thrombin aptamer-functionalized bioanode and a bilirubin oxidase/lysozyme aptamer-functionalized biocathode. The sensor operates on the principle that when thrombin alone is present (input signal (1,0)), the thrombin-binding aptamer layer at the anode impedes the electrode interface, increasing the overpotential for glucose oxidation and reducing the EBFC open circuit voltage. Conversely, the presence of lysozyme alone (input signal (0,1)) results in increased oxygen reduction overpotential at the cathode and decreased cell performance. When both targets are present (input signal (1,1)), a further decrease in voltage output occurs, akin to a NAND logic gate operation. However, this sensor, in its current form, can detect the simultaneous presence of two targets but cannot distinguish between them, indicating scope for improvement. In all these scenarios, the target acts as an inhibitor, placing the sensor in a “signal-off” mode. Taking one step further on these working principles, self-powered sensors in “signal-on” mode can be developed. Li et al. [125] developed a self-powered sensor for the detection of ampicillin. In this work, the anode of the enzymatic biofuel cell was modified with glucose oxidase and aptamer 1 (also a DNA sequence); at the same time, the surface of the silica/gold nanocomposite was modified with the complementary DNA sequence of aptamer 1. Based on DNA complementation technology, the silica/gold nanocomposite material is fixed to the electrode surface and acts as an insulating layer to prevent the contact between glucose and enzymes. When the target is present, due to the immune competition reaction, the aptamer 1 binds to the target, and at the same time, the silica/gold nanocomposite material falls off from the electrode surface, the steric hindrance is reduced, and the cell performance is restored. The degree of improvement in cell performance can reflect the concentration of the target substance and is expected to become a powerful means for on-site detection of food safety.

Overall, the application of nanomaterials to self-powered sensors based on EBFCs has important research significance. Nanomaterials have a high specific surface area and short charge migration distance, which can promote the transmission of electrons between electrodes and electrolytes, thereby increasing the rate and current density of electrochemical reactions. This can enhance the electron transfer between the enzyme and electrode and improve the response speed and signal-to-noise ratio of the sensor to the target substance.

Nanomaterials can serve as cheap carriers and catalysts, replacing traditional precious metals and organic reagents, reducing sensor preparation and operating costs. Nanomaterials can increase the voltage and current output of EBFCs, making the sensors self-powered and reducing the need for external power supplies.

Nanomaterials can serve as carriers or auxiliaries for biocatalysts, improving the stability and selectivity of biocatalysts, as well as their affinity with substrates. For example, nanometals or metal oxides can improve the structure and function of enzymes, and nanocarbon materials can protect enzymes from denaturation and inactivation.

Nanomaterials can control a variety of functions and properties, such as optics, magnetism, mechanics, and biocompatibility, by changing their morphology, structure, and composition. This also realizes the integration of multiple functions, such as photoelectric conversion, magnetic control, drug release, etc., opening up new areas for EBFC applications. For example, nanomaterial-based photobioelectrodes can utilize solar energy to drive bioelectrochemical reactions and improve the energy efficiency of EBFCs. Magnetic bioelectrodes that are based on nanomaterials can use magnetic fields to control the activity and direction of biocatalysts to achieve switching and regulation of EBFCs. Drug release electrodes that are based on nanomaterials can use electrical signals to trigger the release of drugs to achieve the therapeutic function of EBFCs.

## 5. Challenges Faced by EBFC-SPBs

Compared to other types of fuel cells, biofuel cells offer the distinct advantage of operating without the high temperatures that typically lead to the production of nitrogen oxide pollutants, significantly enhancing their safety. Due to their eco-friendly nature, absence of pollution, and the increasing global demand for diverse energy sources, biofuel cells are continually being developed and improved. Moreover, in terms of fuel selection, biofuel cells (BFCs) employ biologically active substances as anode electrocatalysts. This means that virtually any substance that is capable of undergoing biological oxidation can serve as a viable fuel source, in contrast to traditional low-temperature fuel cells, which are generally limited to using H2 or alcohols as fuels. Additionally, the structural design of BFCs is comparatively simpler. Enzymes, known for their high activity and fuel selectivity, facilitate this simplicity. The oxidant and fuel in BFCs do not require separation by an ion-conducting membrane, making the construction of fuel cells with mixed reactants a feasible option. Since biofuel cells use active materials as electrocatalysts, two active materials can be put together, or even only one electrode is required, which makes it possible to miniaturize fuel cells to the microscale [29,129].

In terms of practical applications, BFCs with suitable power density can be used to power sensors. Katz and Willner et al. [130] proposed the idea of self-powered biosensors in which BFCs were used for analyte detection, but the disadvantage is the limited energy supply. With the rapid development of BFC-powered wearable electronic products, it is also affecting human life in areas such as communication, healthcare, entertainment, and other aspects. These wearable electronic devices can be powered by external or physiological fuels from the body, such as tears, lactic acid in sweat, glucose in blood, etc. Implantable biomedical devices have undergone significant changes since the invention of the pacemaker. The arrival of molecular, micron, and nanoscale technologies has led to the miniaturization of all BFC components. The miniaturization of BFCs has greatly expanded their application scope in biomedical devices, such as drug delivery systems, deep brain neurostimulators, glucose biosensors, gastric stimulators, and pacemakers, etc. [131].

Over the past few decades, the biofuel cell sector has witnessed significant research advancements. Yet, it still grapples with challenges such as suboptimal electrochemical performance, incomplete fuel oxidation, a low power output, and poor operational stability. Scientists are focusing on enhancing electrode stability through the integration of materials engineering and biotechnological strategies. This approach aims to create a more conducive environment for enzymes and microorganisms on the electrode surface. Additionally, efforts to improve the electrochemical performance are being explored from various angles. Using electrode materials with high specific surface areas helps increase enzyme loading, enhance enzymatic catalytic activity, and expedite the rate of electron transfer between the enzyme and the electrode. These improvements collectively contribute to an increase in the power and current density of the electrode. Different fuels have different energy densities and oxidation levels, which affect the voltage and current of the battery. In order to improve fuel utilization, multi-enzyme cascade reactions can be used to achieve deep or complete oxidation of the fuel, thereby releasing more electrons. Electrons need to go through multiple steps from the fuel to the anode, and then from the anode to the cathode, including the catalytic reaction of the enzyme, the electron transfer between the enzyme and the electrode, the electron transport within the electrode, and the electron migration within the electrolyte. In order to improve the efficiency of electron transfer, the following methods can be used:

(1) Improve enzyme activity and stability: Through means such as bioengineering or chemical modification, the enzyme’s catalytic ability and ability to resist environmental interference are enhanced.

(2) Promote electron transfer between the enzyme and electrode: By using nanomaterials, electron mediators, conductive polymers, and other means, the distance between the enzyme and the electrode is shortened, the resistance to electron transfer is reduced, and the rate of electron transfer is increased.

(3) Optimize the structure and materials of the electrode: By using electrode materials with a high specific surface area, high conductivity, high porosity, and high catalytic performance, the effective area of the electrode is increased, and the electrochemical performance of the electrode is improved.

(4) Enhance the conductivity of the electrolyte: By using an electrolyte solution with a high concentration, high mobility, and high buffering capacity, the ohmic loss in the electrolyte is reduced, and the pH of the battery is stably maintained.

The oxygen reduction reaction is the main reaction at the battery cathode and the main source of battery voltage loss. The kinetics of the oxygen reduction reaction are affected by factors such as the oxygen solubility, diffusion rate, enzyme catalytic activity, and electron transfer efficiency. To improve the kinetics of oxygen reduction, the following methods can be used:

(a) Increase the supply of oxygen and increase the solubility and diffusion rate of oxygen in the electrolyte by using oxygen pumps, gas–liquid separators, oxygen diffusion electrodes, etc. (b) Select an efficient oxygen reductase by using an oxygen reductase with high affinity, high selectivity, and high stability, such as catalase, double-copper oxygenase, multi-copper oxygenase, etc., to improve the catalytic activity of the oxygen reduction. (c) Optimize the immobilization method of oxygen reductase. By using appropriate fixatives, carriers, connectors, and other means, the spatial configuration and active site of the oxygen reductase are maintained, and the contact area between the oxygen reductase and the electrode and the electron transfer efficiency are increased.

Current research in the domain of enzymatic biofuel cell self-powered biosensors (EBFC-SPBs) primarily focuses on enhancing the stability, detection sensitivity, operational efficiency, and miniaturization of devices. The EBFC-SPBs being designed are primarily used as fundamental analytical and detection tools for identifying abnormally expressed molecules, cells, or proteins in biological systems. However, these tools are not yet capable of providing quantitative information about these abnormally expressed substances, nor can they regulate them effectively. Developing advanced EBFC-SPB sensing modes that can accomplish both detection and regulation is a critical step forward in the evolution of EBFC-SPBs. These novel sensing modes have significantly contributed to the emergence and development of precision control systems that are driven by diagnostic signals.

Therefore, continuous optimization and attempts are required to meet commercial or practical application requirements. Secondly, an EBFC-SPB is currently used for in vitro detection. However, in vivo information is different from in vitro information, so the in vivo detection of EBFC-SPBs needs to be solved urgently. Although some EBFC-SPBs cleverly analyze the relationship between multiple analytes through logic gate control, EBFC-SPBs that can achieve simultaneous quantification of multiple analytes have not yet been reported [132]. Researchers have achieved the dual functions of detection and intelligent regulation through EBFC-SPBs, but they have not yet succeeded in building an EBFC-SPB platform that can perform multiple functions. The microfluidic chip is miniaturized and highly integrated. Combined with EBFC-SPB technology, it will become a more powerful self-powered application platform.

## 6. Outlook and Summary

This article comprehensively reviews the recent applications of nanomaterials in enzyme bioelectrodes and self-powered sensors within enzymatic biofuel cells (EBFCs). Among the nanomaterials that are utilized in EBFCs, carbon-based nanomaterials enhance the specific surface area of electrodes, while metal nanomaterials facilitate electron transfer between enzymes and electrodes. The hybridization of these two types of materials is employed to fabricate three-dimensional (3D) bioelectrodes using conductive nanohybrid materials, which leads to an increase in the current density and output power of EBFCs. Therefore, a future development direction for enzyme electrodes is the preparation of 3D bioelectrodes based on these conductive nanohybrid materials.

EBFCs demonstrate considerable potential in the fields of biosensors and wearable technologies. The ongoing evolution of nanotechnology and its application in enzyme bioelectrodes is poised to revolutionize sensor technology. Firstly, the use of nanomaterials provides enzymatic bioelectrodes with greater specific surface areas and enhanced conductivity, improving the sensitivity and response speed of biosensors. Nanostructured materials can effectively boost the immobilization efficiency of enzymes and increase the number of reaction sites, thereby amplifying the catalytic effect of enzymes. This enhancement will render the biosensor more sensitive in detecting minute quantities of biomolecules, broadening its application scope in healthcare, environmental monitoring, and food safety.

Secondly, the use of enzymatic biofuel cells as self-powered sensors has gained increasing attention. By incorporating nanomaterials into enzymatic biofuel cells, not only is the energy conversion efficiency improved, but they can also be made more compact and portable. This advancement allows biosensors to be self-sufficient in a wider range of environments, reducing their reliance on external power sources and enhancing the practicality and versatility of their applications.

Furthermore, with the emergence of wearable technology, self-powered sensors that are based on nanomaterial-enhanced enzymatic bioelectrodes and EBFCs are expected to become integral components in biomonitoring and medical diagnostics. These sensors could be integrated into smart garments or wearable devices to monitor biomarkers in real time, offering personalized health management solutions.

In summary, the utilization of nanomaterials in enzymatic bioelectrodes and self-powered sensors for EBFCs holds vast potential and is anticipated to play a significant role in medical, environmental, and food safety applications. This development is set to propel further advancements in the field of bioelectrochemistry and pave new research avenues for innovative biosensors and self-powered technologies.

## Data Availability

Not applicable.
